# Supplementary material for: Preexisting Virus-Specific T Lymphocytes-Mediated Enhancement of Adenovirus Infections to Human Blood CD14+ Cells
Source: Viruses. 2019 Feb 13;11(2):154. doi: 10.3390/v11020154 (PMC6409799; doi:10.3390/v11020154)
Supplement: Supplementary file 1 [file viruses-11-00154-s001.pdf]

Supplementary Table 1. Primer sets for qRT-PCR in this study

Gene	Primer	Sequence
CAR	CAR-F	CTGTCAGCCACATATTGAGAT
	CAR-R	CGCCATTTTGAAGACCA
αvβ5	αvβ5-F	CTCATCGTTTCCATTCCAC
	αvβ5-R	CGAGTTTGGTTTTCTGTCTT
SR-A	SR-A-F	CCAGGGACATGGGAATACAA
	SR-A-R	CCAGTGGGACCTCGATCTCC
GM-CSF	GMC-F	GAGGTCCTTGTCCATTCCA
	GMC-R	GCAGATAGCCCATTTCATC
IFN-γ	IFNG-F	AGAGTGTGGAGACCATCAAGGA
	IFNG-R	TGCGTTGGACATTCGAGTCAG
IL-4	IL4-F	CAGTTCTACAGCCACCATGAGAA
	IL4-R	CTCTCTCATGATCGTCTTTAGCCT
B actin	Actb-F	CTGTGCTATGTCGCCCTAGA
	Actb- R	GGAAGGTTGGAAGAGAGCCT

F: forward primer; R: reverse primer.

Supplementary Table 2. The purify of sorted cell populations were determined by FACS assay

Samples	Ad5-seropositive subjects			Ad5-seronegative subjects		
	#1	#2	#3	#4	#5	#6
**percentage of CD19+ B cells in sorted CD3+ T sample (%)**	1.03	0.54	0.83	1.63	0.86	1.20
**percentage of CD3+ T cells in sorted CD3+ T sample (%)**	90.02	97.20	96.57	95.56	96.19	93.53
**percentage of CD19+ B cells in sorted CD19+ sample (%)**	78.78	79.60	80.07	77.98	76.23	79.71
**percentage of CD3+ T cells in sorted CD19+ sample (%)**	13.92	14.50	12.14	15.02	11.10	13.38
**percentage of CD19+ B cells in sorted CD3- sample (%)**	29.64	31.40	30.09	31.77	28.05	29.94
**percentage of CD3+ T cells in sorted CD3- sample (%)**	1.13	0.68	0.75	1.69	0.88	1.16
**percentage of CD19+ B cells in sorted CD19- sample (%)**	0.01	0.12	0.09	0.02	0.01	0.10
**percentage of CD3+ T cells in sorted CD19- sample (%)**	78.04	81.50	79.16	72.97	76.53	80.19
**percentage of CD19+ B cells in sorted CD3-/CD19- sample (%)**	0.42	0.55	0.23	0.45	0.34	0.29
**percentage of CD3+ T cells in sorted CD3-/CD19- sample (%)**	0.17	0.30	0.38	0.11	0.23	0.35
